# Supplementary material for: Differential Paralog-Specific Expression of Multiple Small Subunit Proteins Cause Variations in Rpl42/eL42 Incorporation in Ribosome in Fission Yeast
Source: Cells. 2022 Aug 2;11(15):2381. doi: 10.3390/cells11152381 (PMC9367792; doi:10.3390/cells11152381)
Supplement: Supplementary file 1 [file cells-11-02381-s001.zip › cells-1796889-supplementary.pdf]

cells

Supplementary Materials

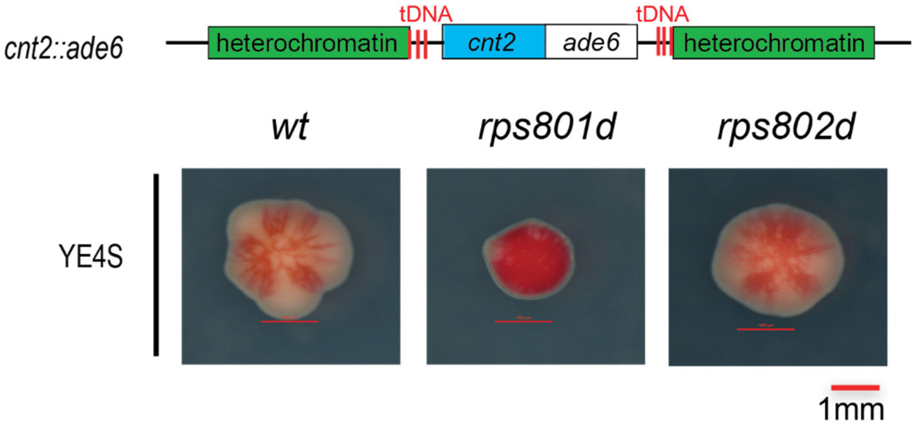

Figure S1. *rps8* paralog deletions show differential colony morphology of CEN-PEV. Cells derived from the red colony among wild type, *rps801d* and *rps802d* were planted at the density of one cell/cm^2 by microscopic manipulation on YE+4S plates, incubated at 25°C for eight days. The representative colony for each strain were shown. Scale bar is 1mm.

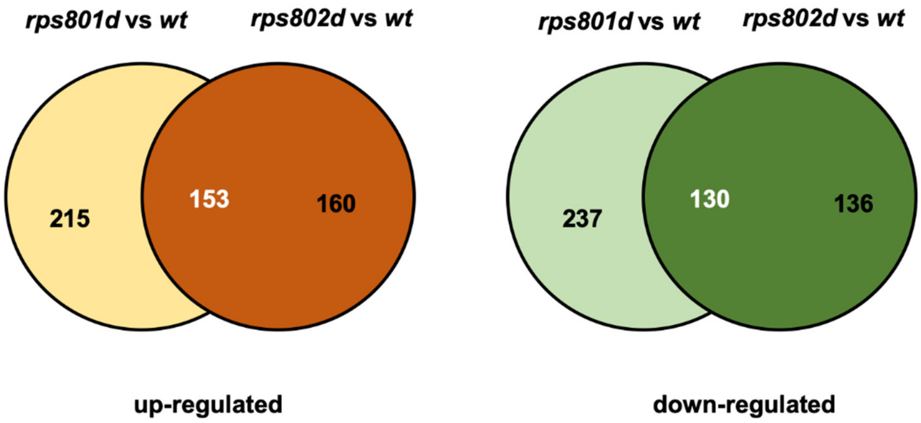

Figure S2. Venn diagram showing the overlap between *rps8* paralog deletions vs wild type. Proteins whose abundance was significantly increased are shown in the left; decreased are shown in the right.

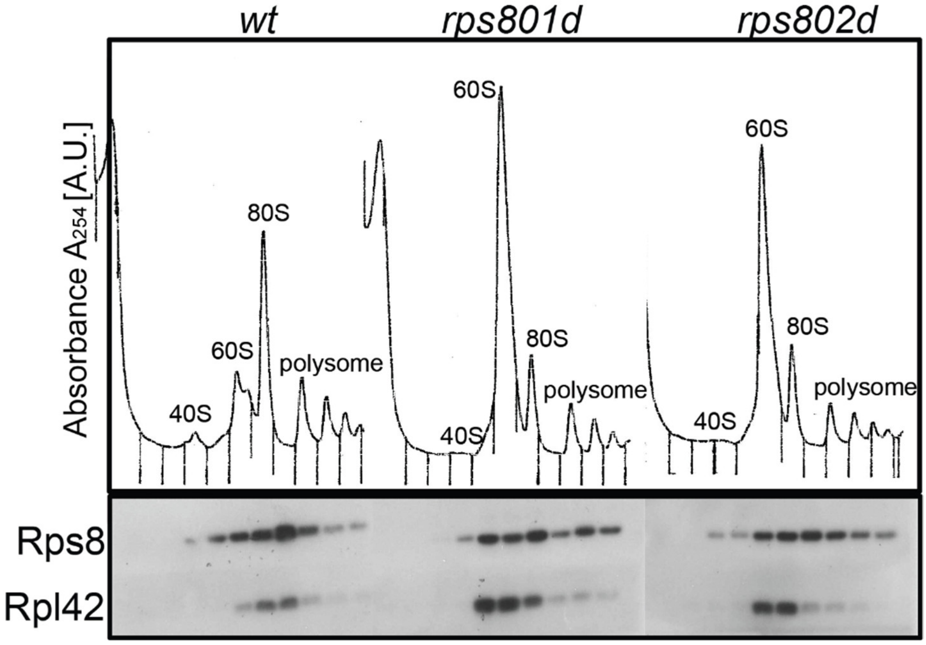

Figure S3. Rpl42/eL42 protein is present in accumulated 60S subunit in *rps8* paralog deletions. Polysome analysis by sucrose-gradient-based centrifugation to assess relative abundance of 40S, 60S, 80S ribosomes and polysomes. And Rpl42/eL42 protein level is detected in each fraction among *wt*, *rps801d* and *rps802d* cells by western blotting.

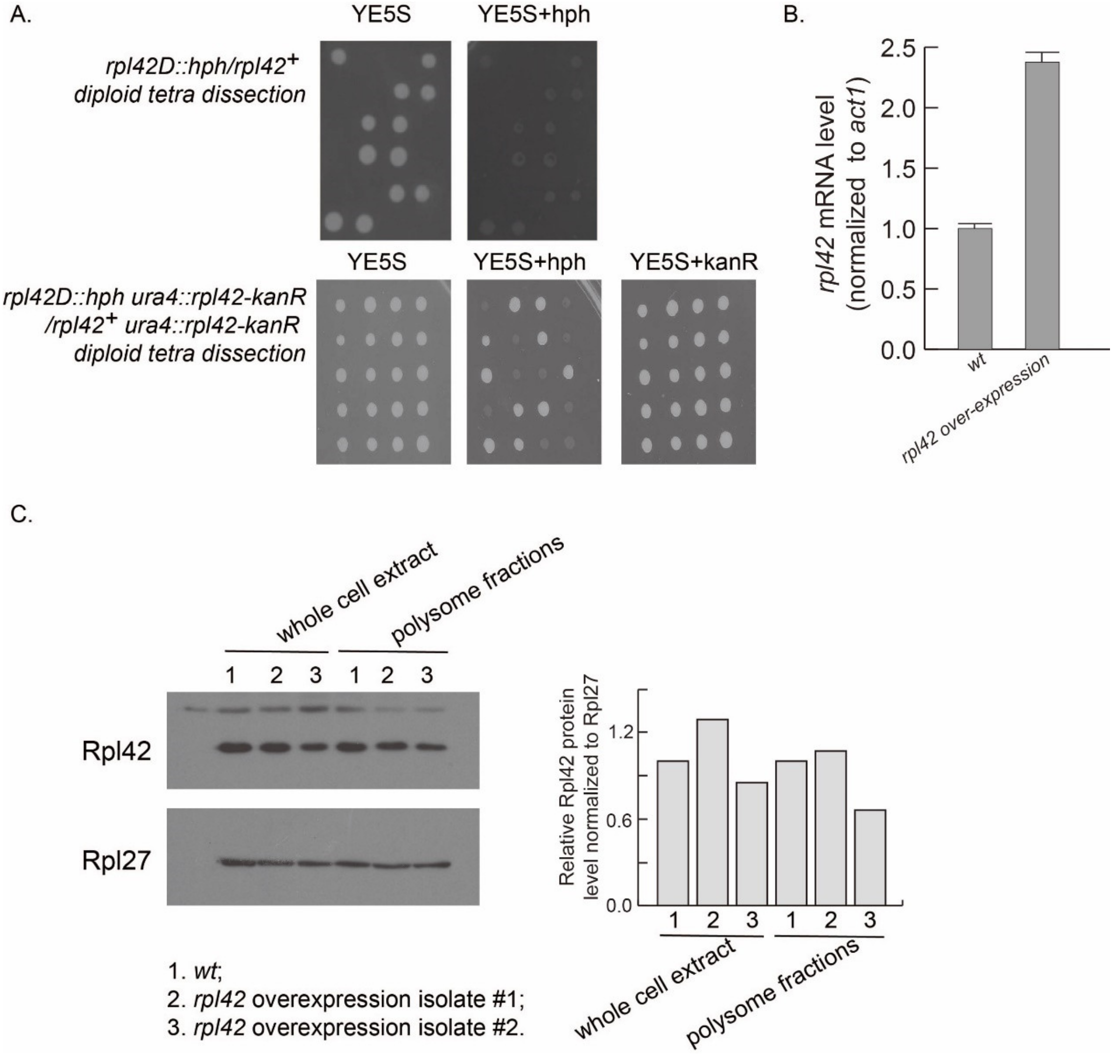

Figure S4. Quantification of *rpl42* mRNA and protein levels when *rpl42* is overexpressed in haploid wild type cells. *rpl42* is an essential gene in haploid wild type cells. B. Quantification of *rpl42* mRNA level by qRT-PCR. C. Quantification of Rpl42/eL42 protein level in whole cell extract and polysome fractions.

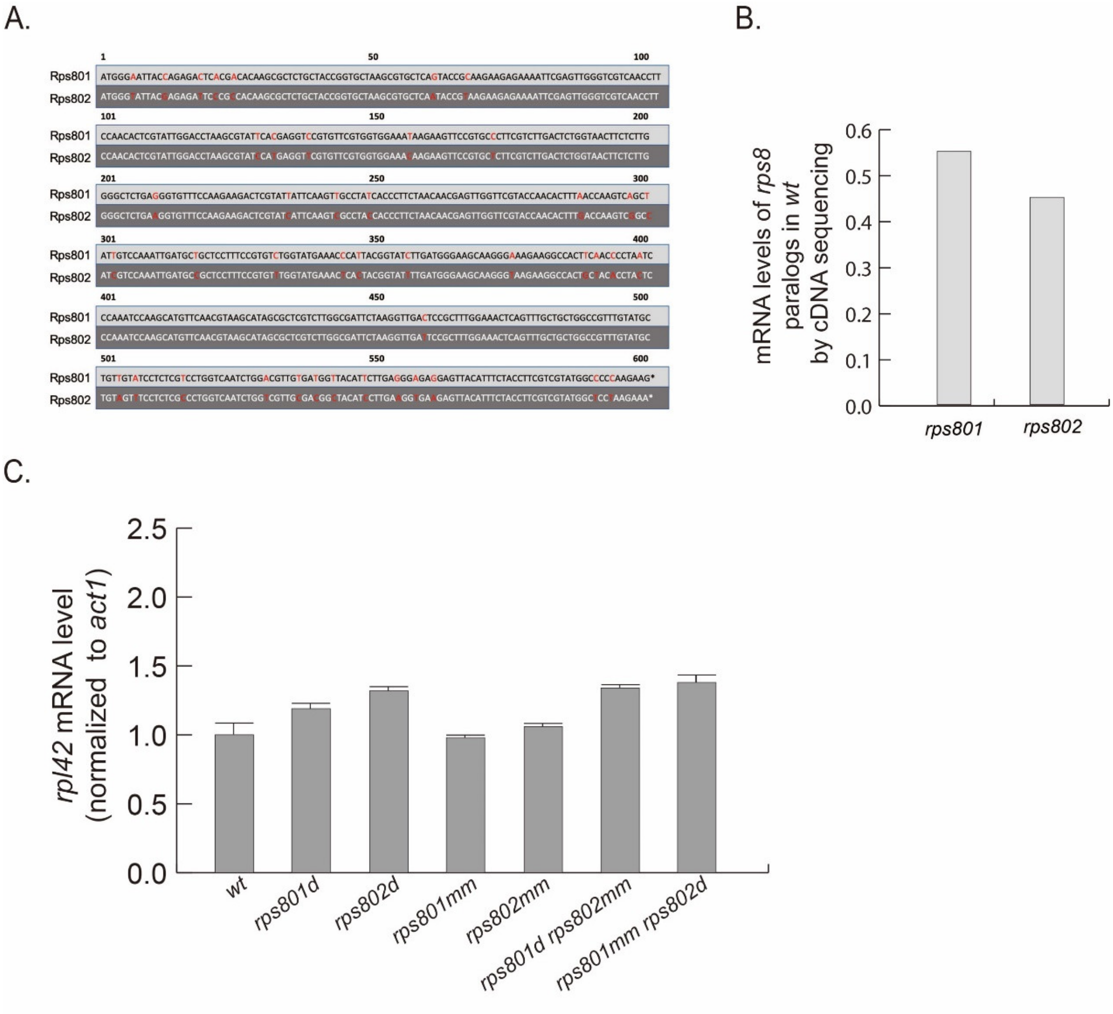

Figure S5. Quantification of Rps8/eS8 paralogs mRNA expression level in wild type cells and quantification of *rpl42* mRNA level in wild type and *rps8* mutants. Comparison of Rps8/eS8 paralogs DNA sequence. B. Quantification of *rps8* paralogs mRNA expression level in wild type cells by cDNA sequencing. C. Quantification of *rpl42* mRNA expression level in wild type and *rps8* mutants by qRT-PCR.

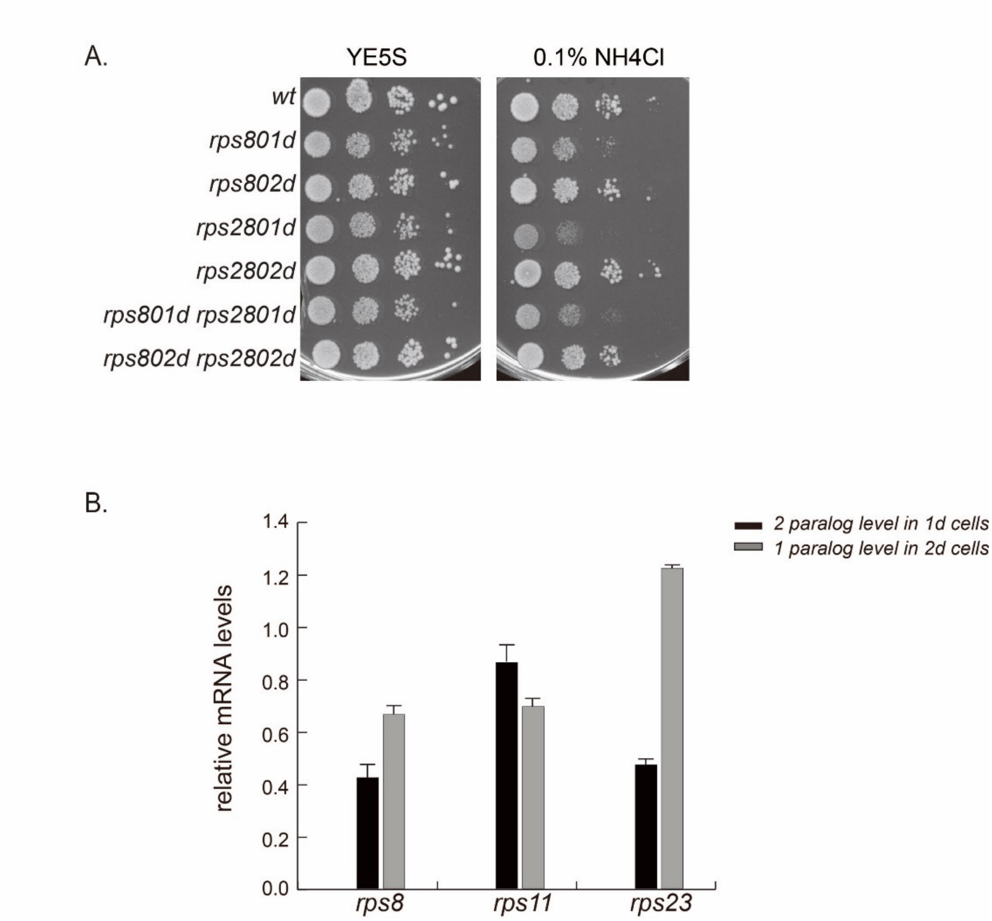

Figure S6. Epistasis analysis between *rps8* paralogs and *rps28* paralogs by spotting assay(A) and the impact of deletion one RP paralog gene on the RP total expression level by qRT-PCR (B).

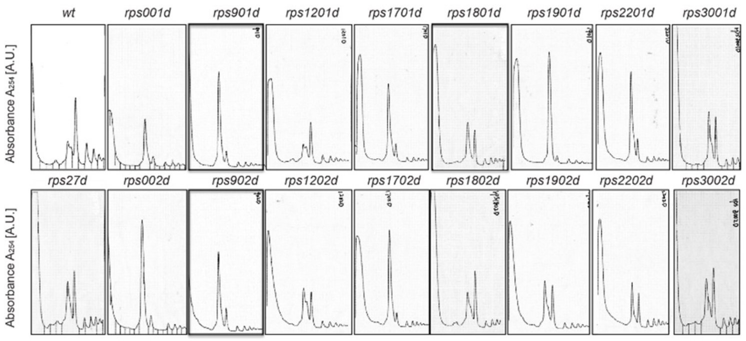

Figure S7. 60S subunits accumulation was observed in other *rps* deletion mutants.

Table S1. Ribosome paralog deletion mutants show multiple paralog-specific phenotypes.

Ribosome proteins	Ribosome paralogs	*cnt2::ade6* PEV	High NH4Cl sensitiv-ity	Cycloheximide sensi-tivity
Rps0/uS2	*Rps001*	WT-like	Resistant	Sensitive
	Rps002	Reduced	Sensitive	Sensitive
Rps1/eS1	*Rps101*	WT-like	NA	NA

	Rps102	NA	NA	NA
Rps4/eS4	*Rps401*	NA	NA	NA
	Rps402	WT-like	NA	NA
	Rps403	WT-like	NA	NA
Rps5/uS7	*Rps5*	NA	NA	NA
	Rps502	WT-like	NA	NA
Rps6/eS6	*Rps601*	NA	NA	NA
	Rps602	NA	NA	NA
Rps8/eS8	*Rps801*	Reduced	Sensitive	Sensitive
	Rps802	WT-like	Resistant	WT-like
Rps9/uS4	*Rps901*	Reduced	Resistant	Sensitive
	Rps902	Reduced	Resistant	WT-like
Rps10/eS10	*Rps1001*	NA	NA	NA
	Rps1002	NA	NA	NA
Rps11/uS17	*Rps1101*	Reduced	Resistant	WT-like
	Rps1102	WT-like	Sensitive	Sensitive
Rps12/eS12	*Rps1201*	WT-like	Resistant	WT-like
	Rps1202	WT-like	Resistant	WT-like
Rps14/uS11	*Rps1401*	NA	NA	NA
	Rps1402	WT-like	NA	NA
Rps15/uS19	*Rps1501*	NA	NA	NA
	Rps1502	Reduced	NA	NA
Rps17/eS17	*Rps1701*	Reduced	Resistant	Sensitive
	Rps1702	Reduced	Resistant	Sensitive
Rps18/uS13	*Rps1801*	WT-like	Resistant	NA
	Rps1802	WT-like	Resistant	NA
Rps19/eS19	*Rps1901*	NA	NA	NA
	Rps1902	WT-like	WT-like	NA
Rps22/uS8	*Rps2201*	WT-like	Resistant	Sensitive
	Rps2202	Reduced	Resistant	WT-like
Rps23/uS12	*Rps23*	Reduced	Sensitive	Sensitive
	Rps2302	WT-like	Resistant	WT-like
Rps24/eS24	*Rps2401*	WT-like	NA	NA
	Rps2402	NA	NA	NA
Rps25/eS25	*Rps2501*	NA	NA	NA
	Rps2502	NA	NA	NA
Rps26/eS26	*Rps2601*	NA	NA	NA
	Rps2602	NA	NA	NA
Rps28/eS28	*Rps2801*	Reduced	Sensitive	Sensitive
	Rps2802	Bi-stable	Resistant	WT-like
Rps30/eS30	*Rps3001*	Reduced	WT-like	WT-like
	Rps3002	Reduced	WT-like	WT-like
Rpl5/uL18	*Rpl501*	WT-like	Resistant	Sensitive
	Rpl502	WT-like	WT-like	WT-like
Rpl8/eL8	*Rpl801*	NA	NA	NA
	Rpl802	NA	NA	NA
	Rpl803	Enhanced	NA	NA
Rpl9/uL6	*Rpl901*	Reduced	Resistant	NA
	Rpl902	Reduced	WT-like	NA
Rpl10/uL16	*Rpl1001*	NA	NA	NA

	Rpl1002	WT-like	NA	NA
Rpl11/uL5	*Rpl1101*	All white	Sensitive	NA
	Rpl1102	WT-like	Sensitive	NA
Rpl12/uL11	*Rpl1201*	WT-like	WT-like	Sensitive
	Rpl1202	WT-like	WT-like	Sensitive
Rpl15/eL15	*Rpl15*	NA	Resistant	NA
	Rpl1502	WT-like	Resistant	NA
Rpl16/uL13	*Rpl1601*	NA	NA	NA
	Rpl1602	NA	NA	NA
	Rpl1603	WT-like	NA	NA
Rpl17/uL22	*Rpl1701*	NA	WT-like	NA
	Rpl1702	WT-like	Resistant	NA
Rpl18/eL18	*Rpl1801*	NA	NA	NA
	Rpl1802	NA	NA	NA
Rpl19/eL19	*Rpl1901*	WT-like	WT-like	NA
	Rpl1902	WT-like	Resistant	NA
Rpl20/eL20	*Rpl2001*	WT-like	Sensitive	NA
	Rpl2002	All white	Resistant	NA
Rpl21/eL21	*Rpl2101*	All white	WT-like	NA
	Rpl2102	NA	WT-like	NA
Rpl24/eL24	*Rpl24*	WT-like	WT-like	WT-like
	Rpl2402	WT-like	WT-like	WT-like
Rpl27/eL27	*Rpl2701*	NA	NA	NA
	Rpl2702	Reduced	NA	NA
Rpl28/uL15	*Rpl2801*	Enhanced	WT-like	WT-like
	Rpl2802	Reduced	Resistant	Sensitive
Rpl30/eL30	*Rpl3001*	NA	NA	NA
	Rpl3002	WT-like	NA	NA
Rpl32/eL32	*Rpl3201*	NA	Resistant	NA
	Rpl3202	NA	WT-like	NA
Rpl36/eL36	*Rpl3601*	Reduced	Resistant	Sensitive
	Rpl3602	Reduced	Resistant	Sensitive
Rpl37/eL37	*Rpl3702*	WT-like	NA	NA
	Rpl3703	NA	NA	NA
Rpl38/eL38	*Rpl3801*	WT-like	Resistant	NA
	Rpl3802	NA	WT-like	NA
Rpl41/eL41	*Rpl4101*	NA	NA	NA
	Rpl4102	NA	NA	NA
Rpl43/eL43	*Rpl4301*	Reduced	Resistant	Sensitive
	Rpl4302	Enhanced	WT-like	WT-like

Note：CEN-PEV phenotypes of the RP paralog deletions were examined by visual inspection of the colony color sectoring morphology. NA: not available; Reduced: colonies showed significant reduction in frequency of color switching; Enhanced: colonies showed significant enhancement in frequency of color switching; Bi-stable: the colonies showed both reduced and enhanced PEV phenotype. The growth rates of the RP paralog deletions on YE media supplied with excess NH4Cl or different concentrations of cycloheximide were assessed with spotting assay analysis. Resistant: the mutant cells grow better than wild type cells; Sensitive: the wild type cells grow better than the mutants.

Table S2. Summary of specific information in different Rps8/eS8 constructs.

Genotype	Gene dosage of Rps8	Protein identity of Rps8	Promoter of Rps8
wt	2	Rps801 and Rps802	P1 and P2

rps801d	1	Rps802	P2
rps802d	1	Rps801	P1
rps801mm (S130A N133T)	2	Rps802	P1 and P2
rps802mm (A130S T133N)	2	Rps801	P1 and P2
rps801mm (S130A N133T) rps802d	1	Rps802	P1
rps802mm (A130S T133N) rps801d	1	Rps801	P2

Table S3. Strain list.

Name	Genotype	Source
446	*h- ade6-210 leu1-32 ura4-D18*	Lab stock
447	*h+ ade6-216 leu1-32 ura4-D18*	Lab stock
LW258	*rps802d::kanR*	This study
LW261	*rps802d::hph*	This study
LW262	*rps801d ::kanR*	This study
LW284	*rps801d ::hph*	This study
LW314	*rps802d ::hph rpl31-GFP ::kanR*	This study
LW316	*rps801d ::hph rpl31-GFP ::kanR*	This study
LW318	*h- rpl31-GFP ::kanR*	This study
LW539	*rpl42$^{+/-}$ ::hph ura4-rpl42 ::kanR diploid #1*	This study
LW540	*rpl42$^{+/-}$ ::hph ura4-rpl42 ::kanR diploid #2*	This study
LW553	*rpl42$^{+/-}$ ::hph diploid*	This study
LW515	*rps801mm(S130A N133T)::hph*	This study
LW454	*rps802mm(A130S T133N)::hph*	This study
LW471	*rps802mm(A130S T133N)::hph rps801d::kanR*	This study
LW537	*rps801mm(S130A N133T)::hph rps802d::kanR*	This study
LW701	*rps801d ::kanR rpl3201-GFP ::kanR*	This study
LW703	*rps802d ::kanR rpl3201-GFP ::kanR*	This study
ZJ-L3201G	*rpl3201-GFP ::kanR*	This study
ZJ-25-8	*rpl42(G51S F58S) ::kanR*	This study
ZJ-25-12	*rps801d ::hph rpl42(G51S F58S) ::kanR*	This study
ZJ-25-6	*rps802d ::hph rpl42(G51S F58S) ::kanR*	This study
ZJ-25-7	*rpl42(K100M A103T) ::kanR*	This study
ZJ-25-15	*rps801d ::hph rpl42(K100M A103T) ::kanR*	This study
ZJ-25-4	*rps802d ::hph rpl42(K100M A103T) ::kanR*	This study
ZJ-DM-5	*rps801d ::hph rps2801d ::kanR*	This study
ZJ-DM-2	*rps802d ::hph rps2802d ::kanR*	This study
LW688	*rps1101d ::kanR rpl42(K100M A103T) ::kanR*	This study
LW689	*rps23d ::kanR rpl42(K100M A103T) ::kanR*	This study
LW691	*rps2302d ::kanR rpl42(K100M A103T) ::kanR*	This study
LW693	*rps1102d ::kanR rpl42(K100M A103T) ::kanR*	This study
LW695	*rps2801d ::kanR rpl42(K100M A103T) ::kanR*	This study
LW697	*rps2802d ::kanR rpl42(K100M A103T) ::kanR*	This study
B08-C06	*rps001d ::kanR cnt2 -ade6 ::nat leu1-32 ura4-D18 ade6D ::hph*	Lab stock
B27-A11	*rps002d ::kanR cnt2 -ade6 ::nat leu1-32 ura4-D18 ade6D ::hph*	Lab stock
B22-A03	*rps101d ::kanR cnt2 -ade6 ::nat leu1-32 ura4-D18 ade6D ::hph*	Lab stock
B03-H10	*rps402d ::kanR cnt2 -ade6 ::nat leu1-32 ura4-D18 ade6D ::hph*	Lab stock
B34-G02	*rps403d ::kanR cnt2 -ade6 ::nat leu1-32 ura4-D18 ade6D ::hph*	Lab stock
B06-G02	*rps502d ::kanR cnt2 -ade6 ::nat leu1-32 ura4-D18 ade6D ::hph*	Lab stock
B30-F11	*rps801d ::kanR cnt2 -ade6 ::nat leu1-32 ura4-D18 ade6D ::hph*	Lab stock

B25-A06	*rps802d ::kanR cnt2 -ade6 ::nat leu1-32 ura4-D18 ade6D ::hph*	Lab stock
B36-G11	*rps901d ::kanR cnt2 -ade6 ::nat leu1-32 ura4-D18 ade6D ::hph*	Lab stock
B29-H01	*rps902d ::kanR cnt2 -ade6 ::nat leu1-32 ura4-D18 ade6D ::hph*	Lab stock
B27-C12	*rps1101d ::kanR cnt2 -ade6 ::nat leu1-32 ura4-D18 ade6D ::hph*	Lab stock
B23-A04	*rps1102d ::kanR cnt2 -ade6 ::nat leu1-32 ura4-D18 ade6D ::hph*	Lab stock
B09-B08	*rps1201d ::kanR cnt2 -ade6 ::nat leu1-32 ura4-D18 ade6D ::hph*	Lab stock
B26-E10	*rps1202d ::kanR cnt2 -ade6 ::nat leu1-32 ura4-D18 ade6D ::hph*	Lab stock
B07-B07	*rps1402d ::kanR cnt2 -ade6 ::nat leu1-32 ura4-D18 ade6D ::hph*	Lab stock
B31-B08	*rps1502d ::kanR cnt2 -ade6 ::nat leu1-32 ura4-D18 ade6D ::hph*	Lab stock
B24-G04	*rps1701d ::kanR cnt2 -ade6 ::nat leu1-32 ura4-D18 ade6D ::hph*	Lab stock
B04-G03	*rps1702d ::kanR cnt2 -ade6 ::nat leu1-32 ura4-D18 ade6D ::hph*	Lab stock
B24-D02	*rps1801d ::kanR cnt2 -ade6 ::nat leu1-32 ura4-D18 ade6D ::hph*	Lab stock
J01-G12	*rps1802d ::kanR cnt2 -ade6 ::nat leu1-32 ura4-D18 ade6D ::hph*	Lab stock
LW-S1901D	*rps1901d ::kanR leu1-32 ura4-D18 ade6 ?*	This study
B33-E07	*rps1902d ::kanR cnt2 -ade6 ::nat leu1-32 ura4-D18 ade6D ::hph*	Lab stock
ZJ-S2201D	*rps2201d ::kanR leu1-32 ura4-D18 ade6 ?*	This study
B31-F02	*rps2202d ::kanR cnt2 -ade6 ::nat leu1-32 ura4-D18 ade6D ::hph*	Lab stock
B28-B01	*rps23d ::kanR cnt2 -ade6 ::nat leu1-32 ura4-D18 ade6D ::hph*	Lab stock
B11-G04	*rps2302d ::kanR cnt2 -ade6 ::nat leu1-32 ura4-D18 ade6D ::hph*	Lab stock
B09-A08	*rps2401d ::kanR cnt2 -ade6 ::nat leu1-32 ura4-D18 ade6D ::hph*	Lab stock
B01-C11	*rps2801d ::kanR cnt2 -ade6 ::nat leu1-32 ura4-D18 ade6D ::hph*	Lab stock
B29-H08	*rps2802d ::kanR cnt2 -ade6 ::nat leu1-32 ura4-D18 ade6D ::hph*	Lab stock
B05-G05	*rps3001d ::kanR cnt2 -ade6 ::nat leu1-32 ura4-D18 ade6D ::hph*	Lab stock
ZJ-S3002D	*rps3002d ::kanR leu1-32 ura4-D18 ade6 ?*	This study
B06-F05	*rpl501d ::kanR cnt2 -ade6 ::nat leu1-32 ura4-D18 ade6D ::hph*	Lab stock
B21-B12	*rpl502d ::kanR cnt2 -ade6 ::nat leu1-32 ura4-D18 ade6D ::hph*	Lab stock
B30-C03	*rpl803d ::kanR cnt2 -ade6 ::nat leu1-32 ura4-D18 ade6D ::hph*	Lab stock
B26-E03	*rpl901d ::kanR cnt2 -ade6 ::nat leu1-32 ura4-D18 ade6D ::hph*	Lab stock
B17-D11	*rpl902d ::kanR cnt2 -ade6 ::nat leu1-32 ura4-D18 ade6D ::hph*	Lab stock
B27-A08	*rpl1002d ::kanR cnt2 -ade6 ::nat leu1-32 ura4-D18 ade6D ::hph*	Lab stock
B22-F07	*rpl1101d ::kanR cnt2 -ade6 ::nat leu1-32 ura4-D18 ade6D ::hph*	Lab stock
B32-B02	*rpl1102d ::kanR cnt2 -ade6 ::nat leu1-32 ura4-D18 ade6D ::hph*	Lab stock
B39-C06	*rpl1201d ::kanR cnt2 -ade6 ::nat leu1-32 ura4-D18 ade6D ::hph*	Lab stock
B20-B01	*rpl1202d ::kanR cnt2 -ade6 ::nat leu1-32 ura4-D18 ade6D ::hph*	Lab stock
B18-H10	*rpl15d ::kanR cnt2 -ade6 ::nat leu1-32 ura4-D18 ade6D ::hph*	Lab stock
B26-B12	*rpl1502d ::kanR cnt2 -ade6 ::nat leu1-32 ura4-D18 ade6D ::hph*	Lab stock
B02-A10	*rpl1603d ::kanR cnt2 -ade6 ::nat leu1-32 ura4-D18 ade6D ::hph*	Lab stock
B36-F08	*rpl1701d ::kanR cnt2 -ade6 ::nat leu1-32 ura4-D18 ade6D ::hph*	Lab stock
B28-B04	*rpl1702d ::kanR cnt2 -ade6 ::nat leu1-32 ura4-D18 ade6D ::hph*	Lab stock
B39-C10	*rpl1901d ::kanR cnt2 -ade6 ::nat leu1-32 ura4-D18 ade6D ::hph*	Lab stock
B23-B02	*rpl1902d ::kanR cnt2 -ade6 ::nat leu1-32 ura4-D18 ade6D ::hph*	Lab stock
B36-G12	*rpl2001d ::kanR cnt2 -ade6 ::nat leu1-32 ura4-D18 ade6D ::hph*	Lab stock
B24-C11	*rpl2002d ::kanR cnt2 -ade6 ::nat leu1-32 ura4-D18 ade6D ::hph*	Lab stock
B28-B12	*rpl2101d ::kanR cnt2 -ade6 ::nat leu1-32 ura4-D18 ade6D ::hph*	Lab stock
B30-H07	*rpl2102d ::kanR cnt2 -ade6 ::nat leu1-32 ura4-D18 ade6D ::hph*	Lab stock
B02-E02	*rpl24d ::kanR cnt2 -ade6 ::nat leu1-32 ura4-D18 ade6D ::hph*	Lab stock
B30-H08	*rpl2402d ::kanR cnt2 -ade6 ::nat leu1-32 ura4-D18 ade6D ::hph*	Lab stock
B03-E03	*rpl2702d ::kanR cnt2 -ade6 ::nat leu1-32 ura4-D18 ade6D ::hph*	Lab stock
B29-E08	*rpl2801d ::kanR cnt2 -ade6 ::nat leu1-32 ura4-D18 ade6D ::hph*	Lab stock
ZJ-L2802D	*rpl2802d ::kanR leu1-32 ura4-D18 ade6 ?*	This study
B04-H09	*rpl3002d ::kanR cnt2 -ade6 ::nat leu1-32 ura4-D18 ade6D ::hph*	Lab stock

B03-B10	*rpl3201d ::kanR cnt2 -ade6 ::nat leu1-32 ura4-D18 ade6D ::hph*	Lab stock
B27-D12	*rpl3202d ::kanR cnt2 -ade6 ::nat leu1-32 ura4-D18 ade6D ::hph*	Lab stock
B21-H11	*rpl3601d ::kanR cnt2 -ade6 ::nat leu1-32 ura4-D18 ade6D ::hph*	Lab stock
B38-A02	*rpl3602d ::kanR cnt2 -ade6 ::nat leu1-32 ura4-D18 ade6D ::hph*	Lab stock
B08-D11	*rpl3702d ::kanR cnt2 -ade6 ::nat leu1-32 ura4-D18 ade6D ::hph*	Lab stock
B01-D06	*rpl3801d ::kanR cnt2 -ade6 ::nat leu1-32 ura4-D18 ade6D ::hph*	Lab stock
LW-L3802D	*rpl3802d ::kanR leu1-32 ura4-D18 ade6 ?*	This study
B08-A07	*rpl4301d ::kanR cnt2 -ade6 ::nat leu1-32 ura4-D18 ade6D ::hph*	Lab stock
B30-C10	*rpl4302d ::kanR cnt2 -ade6 ::nat leu1-32 ura4-D18 ade6D ::hph*	Lab stock
